# An efficient iterative CBCT reconstruction approach using gradient projection sparse reconstruction algorithm

**DOI:** 10.18632/oncotarget.13567

**Published:** 2016-11-24

**Authors:** Heui Chang Lee, Bongyong Song, Jin Sung Kim, James J. Jung, H. Harold Li, Sasa Mutic, Justin C. Park

**Affiliations:** ^1^ Weldon School of Biomedical Engineering, Purdue University, West Lafayette, Indiana, USA; ^2^ J Crayton Pruitt Family Department of Biomedical Engineering, University of Florida, Gainesville, Florida, USA; ^3^ Department of Radiation Medicine and Applied Sciences, University of California San Diego, La Jolla, California, USA; ^4^ Department of Radiation Oncology, Yonsei Cancer Center, Yonsei University College of Medicine, Seoul, Korea; ^5^ Department of Radiation Oncology, University of Florida, Gainesville, Florida, USA; ^6^ Department of Radiation Oncology, Washington University School of Medicine, St. Louis, Missouri, USA

**Keywords:** low-dose imaging, compressed sensing, cone-beam computed tomography (CBCT), gradient projection, backtracking line search

## Abstract

The purpose of this study is to develop a fast and convergence proofed CBCT reconstruction framework based on the compressed sensing theory which not only lowers the imaging dose but also is computationally practicable in the busy clinic. We simplified the original mathematical formulation of gradient projection for sparse reconstruction (GPSR) to minimize the number of forward and backward projections for line search processes at each iteration. GPSR based algorithms generally showed improved image quality over the FDK algorithm especially when only a small number of projection data were available. When there were only 40 projections from 360 degree fan beam geometry, the quality of GPSR based algorithms surpassed FDK algorithm within 10 iterations in terms of the mean squared relative error. Our proposed GPSR algorithm converged as fast as the conventional GPSR with a reasonably low computational complexity. The outcomes demonstrate that the proposed GPSR algorithm is attractive for use in real time applications such as on-line IGRT.

## INTRODUCTION

In recent years, the introduction of cone-beam computed tomography (CBCT) system in radiotherapy procedure has enabled a precise patient positioning prior to the treatment for an on-line targeted radiation delivery [1–3]. The rationale is to utilize the environmental parameters of three-dimensional (3D) CBCT images including the anatomical information of the patient, table setup position, and CT numbers to fine tune the dose delivery plan in real time. However, current protocols for CBCT imaging may not be ideal in terms of dose especially for pediatric patients as patients may be exposed to mildly intense x-rays repetitively over the course of treatment sessions [4].

In order to reduce the imaging dose of CBCT, we need to either 1) minimize the number of x-ray projections based on the anatomy of the patient, and/or 2) reduce the current level of x-ray tube (mAs setting) of each imaging session [5]. In this sense, the conventional standard FDK (Feldkamp, Davis and Kress) reconstruction algorithm can be problematic since the quality of CBCT images is highly prone to noise when only few projection data are available [6]. The degradation appears mainly as 1) streaks (or ripples) induced from large angle differences among projections and 2) white noise induced by sparse x-ray photons in low mAs settings [7–9]. With the recent introduction of compressed sensing theory, it has been proved that noisy and sparsely sampled signals (i.e., lower than the Nyquist rate) can be reconstructed with the use of convex optimization and L1-norm minimization technique [10]. Especially, the total variation (TV) method has been particularly useful in CT reconstruction by exploiting the small variability in x-ray attenuation across the body tissues [8, 9, 11–14]. This theory has offered a promising solution to the CT reconstruction problems in general as it allows to maximally utilize the projection data through an iterative process. Although the application of compressed sensing theory to the CBCT reconstruction seems promising, immediately applying to the clinical practice has been challenging. It was due to the iterative nature of solving the TV-based compressed sensing formulation, which requires calculating multiple forward and backward projections of large datasets over the iterative process [5, 7]. This is well known to be computationally expensive. In order for the compressed sensing based CBCT reconstruction to be practical for its clinical use, the iterations must converge to the desired solution within a clinically feasible timeframe (i.e., within a few minutes). There has been a recent breakthrough where such a major computational bottleneck can be handled with the use of graphics processing unit (GPU) [5, 7, 15–18]. Using the massive parallel processing capability of GPU, the average computational time of several hours or longer can be brought down to below an hour. However, this further needs to be shortened to the order of minutes to be considered clinically feasible. The remaining challenge is in developing an algorithm that handles the computation in an efficient manner while guaranteeing its convergence to the desired 3D image.

In this paper, we propose an iterative but computationally efficient and convergence-proofed image reconstruction algorithm based on the compressed sensing theory. First, we revisit gradient projection for sparse reconstruction (GPSR) algorithm framework of which TV-based compressed sensing formulation can be efficiently solved [16, 19]. Second, we show that a direct solution to the problem requires more than necessary computations for line search processes in each iterative step. We then propose a method that significantly reduces the number of forward projections in each iterative step without compromising the GPSR's convergence rate per iteration. Finally, a comprehensive evaluation of our approach with a numerical phantom as well as a clinical head-and-neck patient sample are presented.

## RESULTS

**Figure 1:**
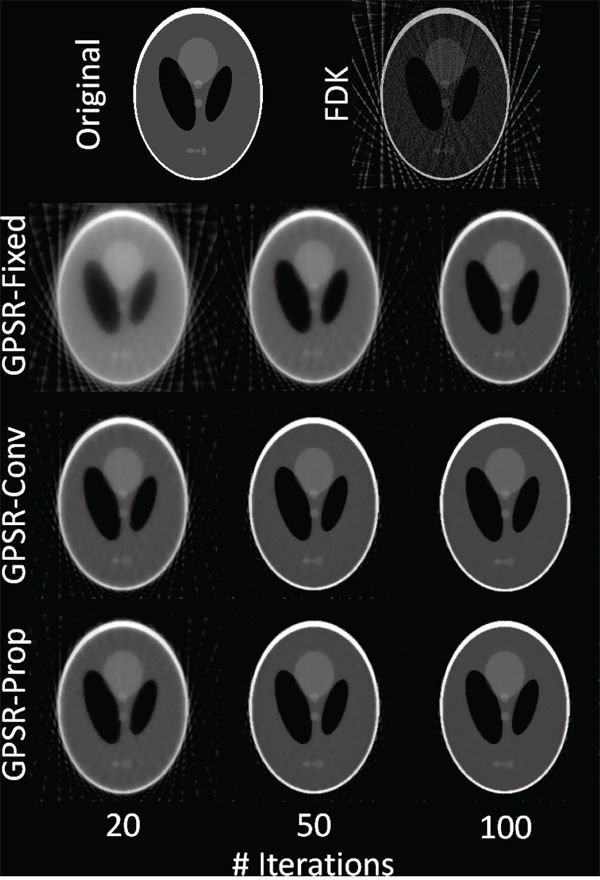
Reconstructed Shepp-Logan phantom images using FDK, GSPR-Fixed, GPSR-Conv, and GPSR-Prop with 20, 50, and 100 iterations A total of 40 projections from 360-degree angle (fan-beam geometry) was used for reconstructions.

Figure 1 demonstrates the image quality of the reconstructed Shepp-Logan phantom with 40 projections using FDK, GPSR-Fixed, GPSR-Conv, and GPSR-Prop. As can be seen, GPSR line search based methods (i.e., GPSR-Conv and GPSR-Prop) resulted in far better image quality than GPSR-Fixed method at every iteration. GPSR-Fixed needed further iterations beyond 100 to converge whereas GPSR-Conv and GPSR-Prop looked to saturate within 100 iterations. Even with 50 iterations, GPSR-Conv and GPSR-Prop were reasonably well reconstructed that the features of the images were easily identifiable. Note that these two methods only differed in their mathematical implementation, and thus the resulting images were exactly the same. However, the computational efficiency of GPSR-Prop largely surpassed that of GPSR-Conv and it is presented in the latter part of this results section.

**Figure 2:**
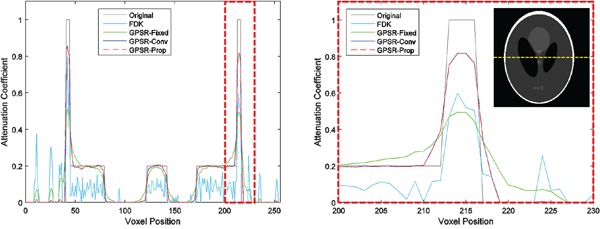
Line profiles of the four algorithms taken from the midline of the Shepp-Logan phantom image which is shown as the yellow dashed line A magnified view of the red dashed region is presented on the right. 40 projections were used. GPSR based algorithms used 50 iterations.

Line profiles in Figure 2 more explicitly illustrate the quality of the reconstructed images by quantitatively assessing by how much each algorithm resembles the original phantom image. FDK was very noisy and prone to fluctuation with 40 projections as the TV term, which enforces sparsity, was naturally omitted in its formulation. GPSR-Fixed was much smoother than FDK, but the weak contrast of the features indicated that more iterations were needed to improve its quality. GPSR line search based methods had a far better feature quality than GPSR-Fixed on the edges of the features. Not only the peaks of the features matched more closely to the original image, but also the side tails dropped to the baseline more sharply. This difference reflects the qualitative difference between GPSR-Fixed and GPSR line search based methods seen in Figure 1.

**Figure 3:**
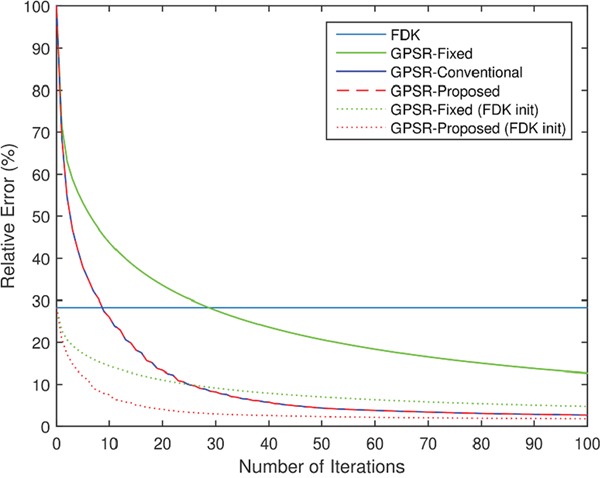
Mean-squared relative error of the four algorithms to the original Shepp-Logan phantom image, as a function of the number of iterations The non-iterative FDK algorithm is presented as a flat line for comparison. 40 projections were used.

Figure 3 shows the mean-squared relative error of the four algorithms as a function of number of iterations. Here, the mean-squared relative error was defined as

$$\text{Relative Error}(\%)=\frac{\|x^{\text{original}}-{x_{k}\|}_{2}^{2}}{\|x^{\text{original}}{\|}_{2}^{2}}\times 100$$

where *x*^original^ is the voxel values of the original Shepp-Logan phantom image. The relative error decreased as GPSR algorithms looped through more iterations. However, there was a clear distinction in the speed of convergence between GPSR-Fixed and GPSR line search based methods. Although a careful choice of *α_k_* minimized this gap as it enhanced the convergence speed of GPSR-Fixed, the gap persisted as the optimal *α_k_* varied in each iteration. With a carefully chosen *α_k_*, GPSR-Fixed outperformed FDK at ∼30 iterations and GPSR-Conv and GPSR-Prop outperformed FDK at ∼10 iterations. Note that this does not mean that GPSR algorithms cannot perform as well as FDK at low iterations. It is possible to set the initial *x*_0_ = *x*^FDK^ so that the performance of GPSR algorithms can improve from that of FDK. Figure 3 also demonstrates by how much this initialization can enhance the convergence speed. GPSR algorithms required no more than 10 iteration with *x*_0_ = *x*^FDK^ to match the performance of 100 iterations with *x*_0_ = *x*^FDK^. The rest of the evaluations in this paper, however, were ran with *x*_0_ = 0_*MN*_ to clearly visualize the importance of iterating with a proper step size.

**Figure 4:**
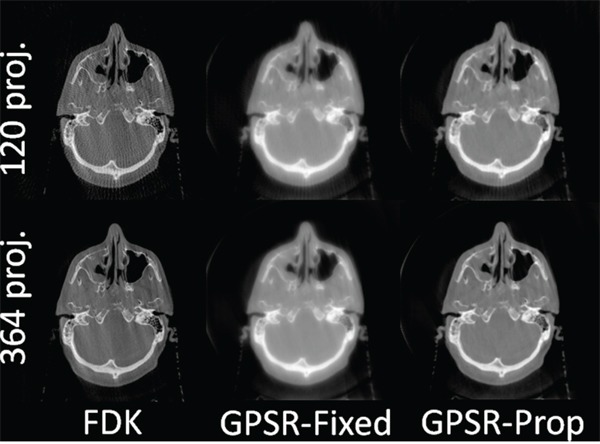
Reconstructed images of a head-and-neck patient sample using 120 and 364 projections GPSR based algorithms used 50 iterations.

Figure 4 demonstrates the reconstructed image quality of a clinically treated head-and-neck patient sample. GPSR-Conv was omitted as it results in exactly same images as GPSR-Prop. Clearly, every method showed far better image quality with 364 projections than with 120 projections. With 120 projections, however, GPSR-Prop outperformed the other methods. The streak artifacts in FDK with 120 projections were not present in GPSR-Prop as they were suppressed by the TV term. Reaffirming that FDK is highly dependent on the number of projections whereas the GPSR methods are more robust, the GPSR-Prop can make use of only fewer projections to reconstruct a reasonably good quality image. Also, GPSR-Prop showed sharper features ratio than GPSR-Fixed with the same number of iterations. Although using more iterations can decrease this gap, doing so also increases the processing time excessively. GPSR-Prop reached close to a fully saturated image with only 50 iterations.

Computational complexities of the GPSR based algorithms for reconstructing the head-and-neck patient sample are compared in Table 1. We see that the number of Radon transforms (i.e., forward/backward projections) and elapsed time per iteration is linearly proportional, which is an evidence that projections are the major bottleneck of the entire computational process. As readers may notice, one forward projection and one backward projection is required for computing the gradient *g*_k_ in each iteration for any types of algorithm. Although GPSR-Prop also needs to perform the same number of function evaluations for the line search process as GPSR-Conv, the simplified Armijo search condition does not require projections to be performed in every function evaluation for finding a proper $\alpha_{k}^{l}$. GPSR-Prop requires one additional forward projection per iteration to obtain *Ap_k_* to perform the backtracking line search. By contrast, GPSR-Conv requires multiple additional projections to be performed as many times as the number of function evaluations for finding a proper $\alpha_{k}^{l}$. Due to the arbitrary nature of $\alpha_{k}^{l}$, the number of projections to be performed varies at each iterations. This resulted in a large standard deviation of the measured time per iteration as seen in Table 1.

If an iterative algorithm with 120 projections can provide a similar image quality as FDK with 364 projections, a dose reduction of 1/3 can be achieved. Not all GPSR algorithms allow us to achieve this lofty goal. Although GPSR-Fixed has a better computational efficiency per iteration than GPSR-Prop, a wise selection of $\alpha_{k}^{l}$ is mandatory and still the convergence rate per iteration is much slower. Likewise, GPSR-Conv is only an inferior version of GPSR-Prop in terms of computational efficiency. Overall, results suggest that GPSR-Prop is a preferable choice among the three presented GPSR algorithms and also over the current clinical standard, FDK, for fast and guaranteed convergence with low-dose projection data.

**Table 1:** A comparison of computational complexities of the three GPSR based algorithms

Image Model		GPSR-Fixed	GPSR-Conv	GPSR-Prop
Head-and-Neck Patient (120 proj.)	Average # of function evaluations per iteration	N/A	6.58	6.58
	Average # of Radon transforms per iteration	2	9.58	3
	Time / Iteration (sec)	6.48 (±0.04)*	30.89 (±0.74)*	9.75 (±0.07)*

Reconstructions took 120 projections from the head-and-neck patient sample using 50 iterations. # of Radon transform operations represents the number of forward/backward projections. * denotes 95% confidence interval.

## DISCUSSION

Up to date, implementation of compressed sensing based CBCT reconstruction onto a real clinical setting has been limited since the solution to its mathematical formulation is numerical rather than analytical [5, 7, 16–18, 20]. A complete single iteration of numerical analysis involves at least one forward and backward projection in addition to other computational processes regardless of the type of algorithm. Although significant amount of computational time can be saved by parallelizing forward and backward projection functions with a GPU, still, majority (>80%) of the time spent to reconstruct a compressed sensing based CBCT is on calculating the projections. Therefore, an algorithm that not only enforces faster convergence, but also minimizes the number of projections at each iterative process is desirable.

Our proposed GPSR approach involves 1) one forward and backward projection which are the minimum requirement for solving any iterative CBCT reconstruction problems and 2) one extra forward projection to ensure the convergence of the cost function to the global minimum. Using the GPU, the computational time measured within each iteration was 9.75 seconds on average, which was approximately three times faster than the original implementation of GPSR (i.e., GPSR-Conv). This shows that our proposed GPSR is computationally efficient and fast while ensuring convergence. GPU implementation accelerated the speed by approximately six times (mean reconstruction time per iteration using Intel i7 CPU was 55.3 sec for GPSR-Prop) as major matrix operations were taken care by hundreds of threads. This synergy between the efficient algorithm and the fast implementation reduced the reconstruction time to within several minutes with 50 iterations. Note that the difference in computational complexity between a CPU and GPU can differ depending on the number of cores and threads that can handle processes in parallel.

One of the main challenges to optimize the image quality of GPSR algorithms is on choosing an optimal regularization parameter *λ*. *λ* must be carefully chosen considering the system parameters such as the type of projections (e.g., distance-driven, ray/voxel driven), scan settings such as the x-ray current (mAs) or number of projections, and other unknown anatomical structures of the patient. While attempting to find an optimal value for this regularization parameter, we observed the noise-contrast tradeoff. The larger the value of *λ*, more weight was kept on the regularization term thereby suppressing the sharpness and making images blurrier. By contrast, when *λ* was assigned too small the regularization effect became too minimal to suppress the noise effectively. We have previously observed that a higher *λ* is desirable for fewer projections to suppress streak artifacts whereas a lower *λ* is desirable for large number of projections to prevent blurring the image [16]. In our study, empirically chosen *λ* for reconstructing 40 projections of the Shepp-Logan phantom was 10 whereas 120 projections of the head-and-neck patient was 1, which well reflects the theoretical expectation. There has been a number of studies and discussions to systematically choose *λ*, but more innovations are required to properly select *λ* in an automated and robust manner while considering aforementioned factors. We expect to benefit from such automation which will improve our algorithm to a more clinically viable solution.

We anticipate that our algorithm can also be useful in four-dimensional (4D) CBCT reconstruction in which a number of streak artifacts are commonly presented due to the lack of projection datasets from a phase/amplitude-wise sorting process. If combined with properly modified cost functions which suits the model of 4D CBCT, we expect that our approach will achieve an improved 4D CBCT quality in a clinically feasible timeframe. However, our algorithm will need further validations for clinical use such as: objective image registration accuracy with planning CT compared with the clinical FDK, implementation on half-fan scans (e.g., thorax, pelvic scans, etc.), and stability of CT numbers for various situations.

### Conclusion

We have presented an efficient and fast way to implement low-dose CBCT reconstruction algorithm using gradient projection for sparse reconstruction approach. Our algorithm provides a solution to minimize the constrained convex CBCT reconstruction cost function through a gradient descent algorithm along with the backtracking line search to find a proper step size in its descent direction. The proposed GPSR approach provides 1) guaranteed convergence to the desired CBCT image which is very important for clinical applications, and 2) fast convergence to a desired solution with lower complexity per iteration, which greatly improves the practical value of the algorithm. With further validations, we envision that our proposed GPSR algorithm will be useful in a real clinical environment such as IGRT, by offering a significant dose reduction from the current clinical standard.

## MATERIALS AND METHODS

### Total variation based iterative CBCT reconstruction

An iterative image reconstruction technique takes either an algebraic approach or a statistical approach. Algebraic reconstruction technique (ART) formulates the following algebraic equation using the x-ray projection data and solves using iterative techniques:

(1) Ax-b=0

where *x* ∈ *R^MN^* denotes the unknown CBCT volume image, *A* ∈ *R^MN × LP^* denotes the forward projection matrix (i.e., Radon transform operator), and *b* ∈ *R^LP^* is the measured projections data. In TV based low-dose CBCT application, the proposed problem is to solve the following constrained convex optimization problem of the form [5, 7–9, 16]:

(2) $$\underset{x}{\min}f(x)=\|Ax-{b\|}_{2}^{2}+\lambda TV(x)\;s.t.x\geq 0$$

where *λ* = regularization constant, and *TV* = Total Variation (TV) regularization term.

The TV term used in this study across *i*, *j*, and *k* axis is defined by

(3) $$TV\left\{x\left(i,j,k\right)\right\}=\sum_{i,j,k}\sqrt{{\left[x(i+1,j,k)-x(i,j,k)\right]}^{2}+{\left[x(i,j+1,k)-x(i,j,k)\right]}^{2}+{\left[x(i,j,k+1)-x(i,j,k)\right]}^{2}}$$

In its form, the first term (fidelity term) enforces fidelity of *x* to the measured projections data and the second term (regularization term) promotes sparsity inherent in the x-ray attenuation characteristics of human body (i.e., the CBCT volume image).

Popular algorithms which have been proposed so far are based on minimizing the fidelity and regularization constraint terms in a separate manner [5, 7–9]. In other words the general framework is based on a two-step procedure, where the fidelity term is first minimized via an ART type algorithm with an arbitrary constant step size, then a solution with the minimal total variation is searched through an optimization process. Such algorithms are likely to converge slowly depending on arbitrary relaxation parameters which are assigned in the ART process [16]. In separate, our intuition is to develop an algorithm which handles fidelity and regularization constraints in a combined manner so as to seek for an answer to the TV-based compressed sensing formulation in a much less number of iteration steps.

### Gradient projection for sparse reconstruction (GPSR) algorithm

Here, we used a gradient projection for sparse reconstruction (GPSR) algorithm [19] that iteratively seeks a solution to Equation (2) in the projected gradient direction while enforcing non-negativity of the found solution. That is, we solved Equation (2) iteratively using

(4) $$x_{k+1}={\left[x_{k}-\alpha_{k}p_{k}\right]}^{+}\text{ where }{\left[*\right]}^{+}=\text{max}\left[*,0\right]$$

and

(5) $$\begin{array}{c} p_{k}=g_{k}\text{ if }g_{k}\leq 0\text{ or }x_{k}\geq 0\\ p_{}=0\text{ otherwise } \end{array}$$

where *α_k_* = step size at iteration *k*, and *p_k_* = projected gradient of function *f(x_k_)*. Here, *g_k_* is the gradient of *f(x_k_)* defined as

(6) $$g_{k}=2A^{T}\left(Ax_{k}-b\right)+\lambda \nabla TV\left(x_{k}\right)$$

In iteratively solving Equation (4), the speed of convergence is entirely dependent on choosing a proper “step-size” *α_k_* in each iteration. Note that the lesser the number of iterations used to find the optimal *x_k+1_* in each iteration, the smaller the amount of time needed for calculating projections *A* and *A^T^*, which are computationally very expensive.

There are several approaches in choosing an appropriate *α_k_* including: a fixed *α_k_* throughout, and a backtracking line-search method that satisfies the Armijo condition. The first method is not trivial in finding an optimal value as the convergence speed and the final image quality are inversely proportional. The second method is popular and guarantees a monotonic convergence, but requires a large computational burden to solve for *α_k_* [21]. We show how this process can be simplified.

Here, we searched each iterative value *x_k_* along the gradient and performed the backtracking line search until a sufficient decrease in the objective function *f(x_k_)* was observed. The initial $\alpha_{k}^{0}$ was chosen as a constant and multiplied by a constant *β* until the line search condition was met.

The backtracking line search rule is governed by the following equation

(7) $$f\left(x_{k}-\alpha_{k}^{l}p_{k}\right)\leq f\left(x_{k}\right)-\delta \alpha_{k}^{l}g_{k}{}^{T}p_{k}$$

which is also called the Armijo condition [5, 7, 16].

**Figure 5:**
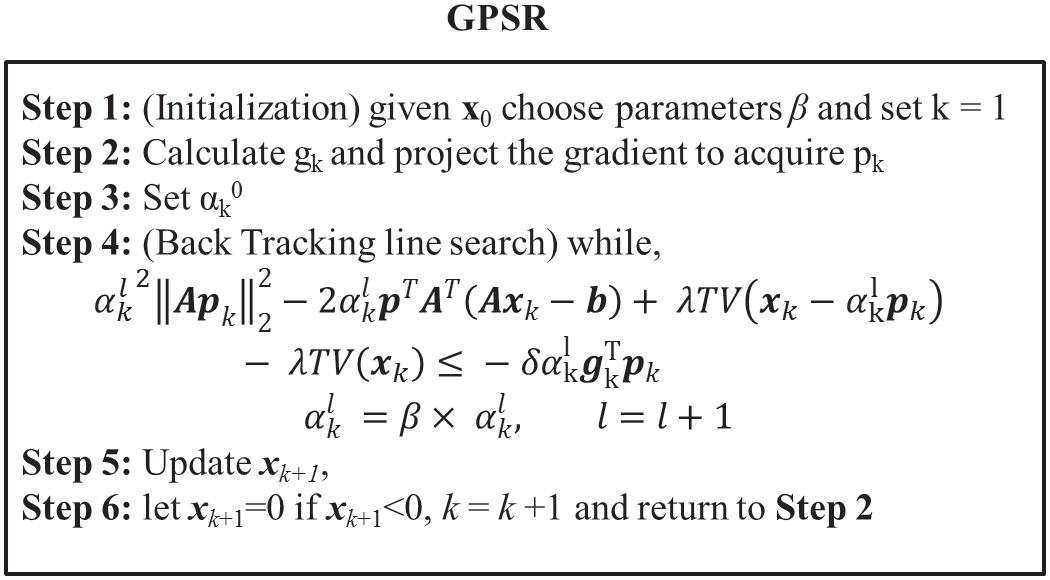
Illustration of computational processes required at each iteration for the proposed GPSR algorithm

However, in order to evaluate the objective function $f(x_{k}-\alpha_{k}^{l}p_{k})$, the Radon transform operator *A* which is the most time consuming operation has to be calculated each and every time while applying $\alpha_{k}^{l}$ value to the gradient. In order to avoid this computational burden, the line search condition (7) was simplified in order to minimize the operation *A*. After modification, the equation shortens to

(8) $$\alpha_{k}^{l}{}^{2}{\left\|Ap_{k}\right\|}_{2}^{2}-2\alpha_{k}^{l}p_{k}{}^{T}A^{T}(Ax_{k}-b)+\lambda TV(x_{k}-\alpha_{k}^{l}p_{k})-\lambda \text{TV}(x_{k})\leq -\delta \alpha_{k}^{l}g_{k}{}^{T}p_{k}$$

in such that the projection operator *A* is carried out only once prior to executing the backtracking line search and not every time it loops through the line search for a proper step size $\alpha_{k}^{l}$. The derivation of Equation (8) is illustrated in the Appendix. A step-by-step algorithm of the proposed GPSR approach is outlined in Figure 5.

### Evaluation of the reconstruction algorithms

To evaluate the performance of our proposed algorithm, we used the well-known Shepp-Logan phantom to compare the convergence rate and computational cost between the three GPSR methods: 1) fixed step-size (GPSR-Fixed), 2) un-modified conventional scheme (GPSR-Conv), and 3) proposed approach (GPSR-Prop). FDK method was also added for comparison to visualize the improvement in image quality from the current clinical standard. In total, 40 projections were acquired across 360 degrees. On evaluating the performance in detail, we have comparatively measured the 1) image quality, 2) attenuation coefficient profile and 3) relative error of the reconstructed image to the original image.

For further evaluation, we used CBCT projection data of a clinically-treated head-and-neck patient acquired from the TrueBeam™ system (Varian Medical Systems, Palo Alto, CA). In total, the data had 364 projections from a 200-degree rotation. The imager had 1024×768 pixels with 0.388×0.388-mm resolution and this was down-sampled to 512×384 pixels with 0.776×0.776-mm resolution through a 2 by 2 binning process. Evenly spaced angles were sub-sampled to emulate varying numbers of projections for image reconstruction. For the patient case, the entire code was structured and implemented in C with CUDA programming environment (NVIDIA, Santa Clara, CA) to utilize the massive parallel computational capability of GPU hardware. In short, the most time consuming operations such as the forward projection, back projection and vector operations were parallelized by assigning each detector pixel values and image voxel values as threads in a GPU.

Parameter values we employed for the evaluations are as follows: *λ* =10 (Phantom) / 1 (Patient), *β* = 0.7, and *δ* = 0.02. The initial voxel values of the unknown CBCT volume image were set to zero (i.e., *x*_0_ = 0_*MN*_, where *MN* is the size of the matrix *x*_k_). For a fair comparison, the initial step size $\alpha_{k}^{0}$ was chosen empirically to make sure that GPSR-Fixed converge to the global minimum and that GPSR-Conv does not fall into excessively many loops within each iteration. GPSR-Prop did not depend on $\alpha_{k}^{0}$ as long as it was chosen sufficiently large.

## Appendix: derivation of equation (8)

By substituting Equation (2) into Equation (7), we have

(A1) $$\begin{array}{l} {}[A(x_{k}-\alpha_{k}^{l}p_{k})-b]T[A(x_{k}-\alpha_{k}^{l}p_{k})-b]+\lambda TV(x_{k}-\alpha_{k}^{l}p_{k})\leq \\ {}[Ax_{k}-b]T[Ax_{k}-b]+\lambda TV(x_{k})-\delta \alpha_{k}^{l}g_{k}{}^{T}p_{k} \end{array}$$

Then, by expanding Equation (A1),

(A2) $$\begin{array}{l} (x_{k}-\alpha_{k}^{l}p_{k})TA^{T}A(x_{k}-\alpha_{k}^{l}p_{k})-2(x_{k}-\alpha_{k}^{l}p_{k}{)}^{T}A^{T}b+b^{T}b+\lambda TV(x_{k}-\alpha_{k}^{l}p_{k})\leq \\ x_{k}{}^{T}A^{T}Ax_{k}-2x_{k}{}^{T}A^{T}b+b^{T}b+\lambda TV(x_{k})-\delta \alpha_{k}^{l}g_{k}{}^{T}p_{k} \end{array}$$

which further expands into

(A3) $$\begin{array}{l} \alpha_{k}^{l}{}^{2}{\left\|Ap_{k}\right\|}_{2}^{2}+x_{k}{}^{T}A^{T}Ax_{k}\;-2\alpha_{k}^{l}p_{k}{}^{T}A^{T}Ax_{k}-2(x_{k}-\alpha_{k}^{l}p_{k}{)}^{T}A^{T}b+b^{T}b+\lambda TV(x_{k}-\alpha_{k}^{l}p_{k})\leq \\ x_{k}{}^{T}A^{T}Ax_{k}-2x_{k}{}^{T}A^{T}b+b^{T}b+\lambda TV(x_{k})-\delta \alpha_{k}^{l}g_{k}{}^{T}p_{k} \end{array}$$

Finally, by eliminating and combining the common terms we have

(A4) $$\alpha_{k}^{l}{}^{2}{\left\|Ap_{k}\right\|}_{2}^{2}-2\alpha_{k}^{l}p_{k}{}^{T}A^{T}(Ax_{k}-b)+\lambda TV(x_{k}-\alpha_{k}^{l}p_{k})-\lambda TV(x_{k})\leq -\delta \alpha_{k}^{l}g_{k}{}^{T}p_{k}$$

which is Equation (8)
